# Status, quality and specific needs of Zika virus (ZIKV) diagnostic capacity and capability in National Reference Laboratories for arboviruses in 30 EU/EEA countries, May 2016

**DOI:** 10.2807/1560-7917.ES.2017.22.36.30609

**Published:** 2017-09-07

**Authors:** Ramona Mögling, Hervé Zeller, Joana Revez, Marion Koopmans, ZIKV reference laboratory group, Chantal Reusken

**Affiliations:** 1 Department of Viroscience, Erasmus University Medical Centre, Rotterdam, the Netherlands; 2 European Centre for Disease Prevention and Control, Stockholm, Sweden; 3 Netherlands Centre for Infectious Disease Control, Bilthoven, the Netherlands; 4 The members of the ZIKV reference laboratory group are listed at the end of the article

**Keywords:** vector-borne infections, laboratory preparedness and response, Zika virus, ZIKV, emerging diseases, re-emerging diseases, diagnostic

## Abstract

With international travel, Zika virus (ZIKV) is introduced to Europe regularly. A country's ability to robustly detect ZIKV introduction and local transmission is important to minimise the risk for a ZIKV outbreak. Therefore, sufficient expertise and diagnostic capacity and capability are required in European laboratories. To assess the capacity, quality, operational specifics (guidelines and algorithms), technical and interpretation issues and other possible difficulties that were related to ZIKV diagnostics in European countries, a questionnaire was conducted among national reference laboratories in 30 countries in the European Union/European Economic Area (EU/EEA) in May 2016. While the coverage and capacity of ZIKV diagnostics in the EU/EEA national reference laboratories were found to be adequate, the assessment of the quality and needs indicated several crucial points of improvement that will need support at national and EU/EEA level to improve ZIKV preparedness, response and EU/EEA ZIKV surveillance activities.

## Introduction

Zika virus (ZIKV) infections were historically not considered a significant public health concern [1]. However, in the year following its first autochthonous transmission in the Americas in 2015, ZIKV has been linked to severe congenital anomalies in newborns and to other neurological disorders such as Guillain–Barré syndrome (GBS) [2]. The World Health Organization (WHO) declared the cluster of microcephaly cases and other neurological disorders possibly associated with ZIKV a Public Health Emergency of International Concern (PHEIC) on 1 February 2016 [3]. By the end of 2016, the unprecedented ZIKV outbreak affected 48 countries and territories in the Americas with more than half a million human cases [4]. The PHEIC was declared over on 18 November 2016 with the argument that ZIKV-associated congenital syndrome was considered to be a long-term public health challenge requiring a long-term commitment to control and prevent [4,5].

ZIKV is a mosquito-borne virus which belongs to the genus *Flavivirus*, family *Flaviviridae* [6]. Its genome can be detected in biological samples by reverse transcription PCR (RT-PCR) and the virus can be isolated in cell culture. However, viraemia is typically short-lived (3–7 days after onset of symptoms) [6-8], although an increased window of detection has been observed using urine (up to 20 days after onset of symptoms) [8] or whole blood (up to 100 days after onset of symptoms) [9]. Consequently, the full spectrum of diagnostics includes serology, which is complex owing to extensive cross-reactivity with antibodies triggered by other flaviviral infections and/or vaccination [10]. The majority of ZIKV infections are asymptomatic which complicates retracing the course of infection and increases the dependency on serology to confirm an infection [10]. This is in particular an issue for pregnant women for whom correct diagnosis of a ZIKV infection, even if asymptomatic, is imperative [2,11,12].

International travel and outbreaks of ZIKV in European overseas countries and territories are responsible for the regular introduction of ZIKV to Europe [13-16]. A risk assessment by the WHO Regional Office for Europe (WHO/Europe) indicated that the risk for an outbreak with ZIKV in Europe should not be underestimated, in particular in countries with established presence of the vectors *Aedes aegypti* and *Ae. albopictus* [16,17]. A country’s ability to robustly detect ZIKV introduction and local transmission is important to minimise the risk for a ZIKV outbreak. Therefore sufficient expertise and diagnostic capacity and capability in European laboratories are required [18].

To map ZIKV expertise and identify diagnostic capacity and capability gaps in Europe during the initial phase of the PHEIC in February 2016, the European Commission (EC) asked the European Centre for Disease Prevention and Control (ECDC) for a rapid assessment of the capacity of laboratories in Europe to detect ZIKV infections and the specific needs for support. The assessment was done by an in-depth questionnaire in May 2016. Here, the outcomes of the assessment are presented and discussed to identify knowledge and technical gaps to strengthen laboratory capacity and quality for ZIKV diagnostics in the European Union/European Economic Area (EU/EEA) and to support ZIKV preparedness and response as well as ZIKV surveillance activities in the EU/EEA. 

## Methods

A questionnaire was designed to address the capacity, quality, operational specifics (guidelines and algorithms), technical and interpretation issues and other possible difficulties related to ZIKV diagnostics. National reference laboratories for ZIKV diagnostics were asked to complete the online questionnaire through the 30 EU/EEA ECDC National Focal Points for Microbiology (NMFPs) [15]. The questionnaire was sent to the NMFPs on 4 May 2016 and closed on 23 May 2016. As most questions in the questionnaire where not obligatory to answer, the sums in the presented data may vary as non-replies are not listed. 

## Results

### In-country presence of ZIKV diagnostics

**Figure 1:**
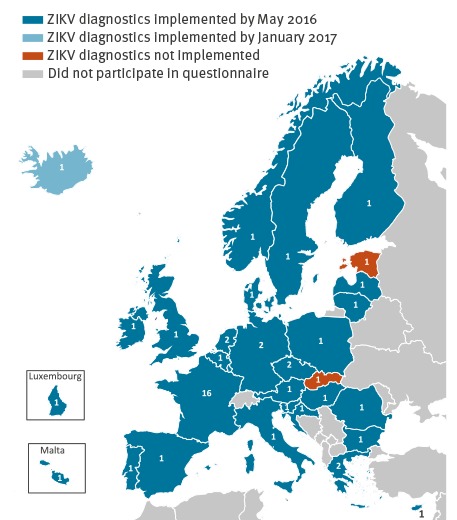
Status of availability of Zika virus diagnostics in EU/EEA countries by May 2016 (n = 49 laboratories)

In total, 49 laboratories from 30 EU/EEA countries completed the questionnaire, of which 44 laboratories in 27 countries indicated that they were conducting ZIKV diagnostics by May 2016 (Figure 1).

EU/EEA: European Union/European Economic Area; NMFP: National Focal Points for Microbiology; ZIKV: Zika virus.

The number of laboratories that participated in the questionnaire on invitation by their country NMFP in the period 4–23 May 2016 are indicated per country. One country indicated in January 2017 that they had implemented ZIKV diagnostics since May 2016. Two countries without implemented ZIKV diagnostics indicated to have access to diagnostics through an agreement with a laboratory in another country.

### Legislation

ZIKV infection was made notifiable in 13 countries by May 2016, and mandatory disease notification was planned for the near future in nine countries. For one country, the notification procedure was not specified. In agreement with the EU classification [19,20], 34 laboratories indicated that their operational biosafety level (BSL) for ZIKV diagnostics was BSL2. Eight laboratories indicated BSL3 as their operational biosafety level, while two laboratories performed ZIKV diagnostics at BSL1. Reasons given by laboratories to deviate from the official level BSL2 were (i) downgrading because diagnosis did not involve high titre virus culture (one laboratory), and (ii) upgrading based on in-house assessment of biosafety and biosecurity issues or for logistical reasons such as aligning ZIKV diagnostics with other flavivirus diagnostic serology and virus isolation (three laboratories). The remaining six laboratories did not indicate reasons for the deviation.

Because of, at the time putative [21], teratogenic effects of ZIKV infection in pregnant women, laboratories conducting ZIKV diagnostics were asked whether special regulations for pregnant employees were in place. In six of the 33 laboratories that answered this question, pregnant employees were not allowed to perform ZIKV diagnostic tests. In three additional laboratories, a restriction for pregnant employees was limited to handling ZIKV virus culture. The remaining laboratories did not have specific guidelines for pregnant employees.

### Sample characteristics

All laboratories conducting ZIKV diagnostics (n = 44, in 27 countries) performed molecular testing, while 33 laboratories in 24 countries also conducted serology and one laboratory performed antigen detection. The laboratories accepted different types of patient samples, including plasma/serum, urine, semen, amniotic fluid, placenta, saliva and nasopharyngeal swabs. All laboratories received serum samples for primary diagnostics, and in addition, clinicians often provided urine as a second sample for testing (42/44). Semen (28/44), saliva (23/44) and nasopharyngeal swabs (15/44) were received to a lesser extent. 

**Figure 2:**
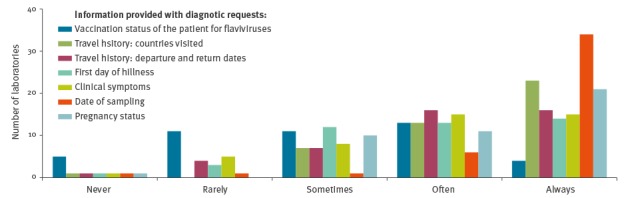
Access of Zika virus diagnostic laboratories to essential background information on patient samples, EU/EEA, May 2016 (n = 44)

For an adequate choice of diagnostic tests to run and for a correct interpretation of results, a minimum of information on the patient history is required [22-24]. Laboratories were asked to indicate on a Likert scale with 1–5 range (1 = never, 5 = always) how often certain essential background information about their patient samples was provided with the diagnostic requests (Figure 2).

EU/EEA: European Union/European Economic Area.

Overall, the provision of the date of sampling was scored the highest (40/44 laboratories scored this in category ‘often’ or ‘always’), while the flavivirus vaccination status scored the lowest (17/44 scored it as ‘often’ or ‘always’). Other data essential for choice of testing algorithm and test interpretation such as ‘first day of illness’, ‘country and dates of travel’, ‘clinical symptoms’ and (in case of women) ‘pregnancy status’ were provided with requests in 27 to 36 of the 44 laboratories.

Most laboratories (26/44) indicated that they could directly contact physicians to ask for more information about the requested samples or look up record sheets and laboratory reports. However, in practice the feasibility of this strongly depended on the daily sample load.

### Molecular diagnostics

Twenty-one laboratories that conducted ZIKV molecular detection only used commercially available tests, 14 laboratories only in-house tests, while nine laboratories indicated using both. The RealStar Zika virus RT-PCR kit by Altona (Hamburg, Germany) diagnostics was the most widely used commercial test (24/30). The most applied in-house RT-PCRs previously described in literature were by Lanciotti et al. (5/23) [25] and Faye et al. (9/23) [26]. Primary RT-PCRs were mostly carried out with a single ZIKV genome target (42/44). However, two laboratories performed a multiplex PCR targeting multiple viruses which were not specified further.

Positive results were confirmed independently in 16 of 44 laboratories. This was done either by a second RT-PCR targeting a different genome segment (8/16 laboratories) or through sequencing (5/16). Forty-three of 44 laboratories used a positive control, which was obtained through the European Virus Archive (https://www.european-virus-archive.com) in six, through the Robert Koch Institute (via former ENIVD expert laboratory network) in 10, from own patient materials in 14, or by using synthetic RNA in four of 43 laboratories. Twenty-two laboratories indicated that they used another positive control, but not the specific source.

Eighteen of 43 laboratories used ZIKV strains of the current outbreak in the Americas as a control, while 14 used strains belonging to the original (pre-2015) Asian lineage. African lineage strains were used in 18 laboratories, 10 had access to more than one ZIKV lineage as control.

### Serology

ZIKV serology was conducted in 24 countries and in 33 of 44 laboratories with ZIKV diagnostics all of which had previous experience with serology-based diagnostics for other flaviviruses, notably dengue virus (DENV, 32/33), tick-borne encephalitis virus (TBEV, 25/33), West Nile virus (WNV, 27/33), Japanese encephalitis virus (JEV,20/33) and yellow fever virus (YFV, 21/33). Serology was based on IgM detection only (2/33) or on both ZIKV IgM and IgG (31/33). Ten laboratories in 10 countries were able to assess the presence of ZIKV neutralising antibodies by plaque reduction neutralisation (PRNT) and/or virus neutralisation (VNT) tests. Thirty-one of 33 laboratories carried out commercial serology assays. Laboratories either used the Euroimmun AG Anti-Zika virus ELISA IgM/IgG (12/31), Euroimmun Arboviral Fever Mosaic IF kit (3/31), both assays (3/31) or did not specify which assay they used (13/31). Thirteen of 33 laboratories used in-house assays, including ELISA (2/13) and/or immunofluorescence assays (IFA) (6/13), as well as VNT (7/13) and PRNT (3/13). The majority of these laboratories (12/13) indicated that they obtained their positive control material from own patient samples. Four of 13 laboratories were provided with ZIKV IgM/IgG positive control samples by collaborators.

### Quality assessment

To gain insight in the level of quality control at the laboratories that performed ZIKV diagnostics, the laboratories were asked to specify their level(s) of laboratory accreditation. Analysis at the laboratory level showed that International Organization for Standardization (ISO) 15189 (‘Medical laboratories – requirements for quality and competence’ [27]) was implemented at 27 of the 44 diagnostic laboratories, five laboratories had accreditation ISO 17025 (‘General requirements for the competence of testing and calibration laboratories’ [28]), while two laboratories indicated to have both. Three laboratories indicated ISO 15189 accreditation was pending or planned. Two laboratories were working under accreditation ISO 9001 (‘Quality management systems – requirements’ [29]) while two laboratories indicated that they operated under a national quality accreditation system. Five reference laboratories had no accreditation at all (data not shown).

Analysis at country level showed 10 of 27 countries with reference laboratories with ISO 15189 accreditation, five countries with laboratories with ISO 17025 accreditation, two countries with laboratories with a national accreditation, two countries with laboratories with ISO 9001 accreditation, two countries with laboratories with both ISO 15189 and ISO 17025 accreditation, two countries with laboratories with either ISO 15189 or no accreditation, one country with a laboratory in transition to ISO 15189 and three countries with laboratories without accreditation.

Another quality aspect concerns the extent of validation of the implemented diagnostics, in particular the serology in view of extensive cross-reactivity. The median size of in-house validation panels of confirmed ZIKV and WNV patients was small (Table 1) and serum samples from pregnant women and population panels from ZIKV-endemic regions were lacking in most laboratories (Table 1). Thirty-seven of 44 laboratories indicated that they were willing to share validation data with other laboratories. However, 15 of the 44 ZIKV diagnostic laboratories indicated that their accreditation scheme did not accept validation done elsewhere, while this would be a possible option for 22 of 44 laboratories.

**Table 1:** Median size of different validation panels for Zika virus diagnostic serological tests, EU/EEA, May 2016 (n = 28 laboratories)

	Number of laboratories	Median size of validation panels (1st quartile; 3rd quartile)
Confirmed ZIKV patients	15	10 samples (3; 18.5)
Confirmed DENV patients	18	20 samples (10; 48)
Confirmed WNV patients	10	10 samples (5; 12)
Confirmed CHIKV patients	16	12 samples (9.75; 20)
Confirmed YFV, JEV or TBEV vaccinated	15	20 samples (10; 43)
EBV panel	10	15 samples (10; 45)
CMV panel	10	15 samples (10; 45)
Malaria panel	9	22 samples (10; 34)
Population panel own country	13	50 samples (20; 100)
Population panel endemic region	4	12.5 samples (8.25; 136.25)
Pregnancy panel	7	100 samples (39; 100)

CHIKV: chikungunya virus; CMV: cytomegalovirus; DENV: dengue virus; EBV: Epstein Barr virus; EU/EEA: European Union/European Economic Area; JEV: Japanese encephalitis virus; TBEV: tick-borne encephalitis virus; WNV: West Nile virus; YFV: yellow fever virus; ZIKV: Zika virus.

### Capacity

**Figure 3:**
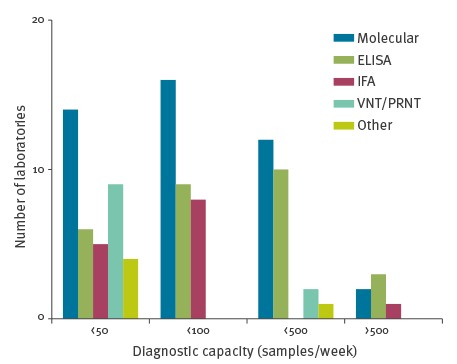
Diagnostic capacity for different types of tests for Zika virus (samples/week) in EU/EEA reference laboratories, May 2016 (n = 44)

ELISA: enzyme-linked immunosorbent assay; EU/EEA: European Union/European Economic Area; IFA: immunofluorescence assay; VNT: virus neutralisation test; PRNT: plaque reduction neutralisation test.

Laboratories were asked to indicate how many diagnostic samples they could process per week for the different types of test that they run (Figure 3).

Diagnostic capacities of individual laboratories differed depending on the type of diagnostic test. In addition, the laboratories were asked how many samples they had processed and determined positive for molecular, IgM, IgG and neutralising antibody testing since 1 January 2016 (Table 2). For 33 of 44 laboratories offering molecular testing, the cumulative number of total requests in the 18-week period covered in the questionnaire (including requests in support of other laboratories) remained below their indicated capacity per week. For 8 of 44 laboratories, the cumulative number of requests was approximately two or three times the indicated weekly capacity, while for three laboratories, it exceeded their capacity (five to 10-fold). For serology, a vast majority of the laboratories had a cumulative number of requests for the 18-week period that was two to three times their weekly capacity.

**Table 2:** Patient samples tested by EU/EEA reference laboratories, January–May 2016 (n = 44)

Type diagnostic test	Tested samples	ZIKV-positive samples
Molecular	7,570	729
IgM antibodies	7,357	396
IgG antibodies	7,205	956
Neutralising antibodies	1,012	191

EU/EEA: European Union/European Economic Area.

Questionnaire period started on 1 January and ended between 4 and 23 May.

Fourteen of 44 laboratories indicated that they supported other laboratories by performing molecular tests for them. Fifteen laboratories supported others with serological testing and 12 laboratories with provision of control materials. The majority of the laboratories (24/29) that had not offered laboratory support until May 2016 indicated that they would be willing to if needed.

### ZIKV testing algorithm

Laboratories mainly implemented the ZIKV testing algorithms either as advised by ECDC (18/44) [30] or by their National Public Health institutes (18/44). The remaining eight laboratories followed, among other algorithms, the ZIKV diagnostic algorithms advised by the Pan American Health Organization (PAHO) [31]. In case necessary background information such as first day of illness was not known, ten of the 44 diagnostic laboratories performed both molecular and serological tests on the available samples, three always carried out serology, two always performed RT-PCR, while three only conducted the tests that were asked for by the clinician. Ten laboratories either asked for more information or an additional sample from the patient for an interpretation based on kinetics. The remaining 16 did not provide that answer. 

Laboratories were also asked if there was a different testing algorithm for asymptomatic pregnant women with putative ZIKV exposure. Ten laboratories indicated that they asked for paired serum samples of asymptomatic pregnant women for serology, while 16 laboratories did both molecular and serological testing on the available samples. Eight laboratories referred to their national ZIKV diagnostic algorithm and one laboratory indicated that asymptomatic patients were never tested regardless of pregnancy status.

### Diagnostic challenges

**Figure 4:**
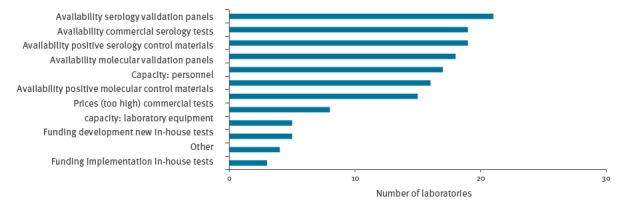
Challenges indicated by EU/EEA laboratories that they were faced with during the implementation of ZIKV diagnostics, May 2016 (n = 44)

Laboratories were asked to describe the main challenges they were faced with during the implementation of ZIKV molecular and/or serological diagnostics. The main indicated obstacles were the availability of a positive control and validation materials for molecular and serological tests, the availability of commercial serological tests and personnel capacity (Figure 4).

## Discussion

In May 2016, there was an EU/EEA-wide coverage for ZIKV molecular diagnostics. Only three countries were without in-country ZIKV molecular diagnostics but all had access to diagnostics through an agreement with a laboratory in another country and two had plans to implement ZIKV diagnostics in the near future. Of these, one country had implemented ZIKV molecular diagnostics by January 2017 (Figure 1). In comparison to a February 2016 snapshot for the EC (data not shown), four more EU/EEA countries had implemented ZIKV molecular diagnostics in May. The coverage for ZIKV serology increased from 17 EU/EEA countries to 24. Access to ZIKV serology is particularly important because the confirmation or ruling out of a ZIKV infection during pregnancy is essential for medical follow-up regarding the teratogenicity of ZIKV [2,11,12]. As an estimated 80% of ZIKV infections are asymptomatic and the genome detection window in serum is short [10], ZIKV diagnosis in pregnant women will often rely on ZIKV antibody detection in paired serum samples.

An important aspect of conducting ZIKV serology is expertise about flaviviruses because the interpretation is complex [10,30,32]. All laboratories conducting ZIKV serology indicated that they had experience with serodiagnostics for at least one other flavivirus. In addition, insight in the specificity and sensitivity of the serology tests used is required for proper test interpretation, but this appeared limited at the time of the questionnaire because adequate validation panels were lacking. Validation data provided by commercial entities typically need to be confirmed in order to be acceptable for an accreditation scheme [27]. Similarly, validation of such assays performed in another laboratory may be insufficient to meet with accreditation requirements. Indeed, the availability of validation panels for serology was indicated as the biggest challenge for implementation of ZIKV diagnostics by 21 of 44 ZIKV diagnostic laboratories. Five of the ZIKV diagnostic laboratories did not have any kind of ISO accreditation, while 17 of 44 laboratories did not have the most relevant ISO 15189 accreditation [27]. Another concern is the broad reliance for ZIKV serology on one or two commercial tests, although it should be noted that in May 2016, the commercial market for ZIKV serology offered very limited options. In December 2016, no commercial serology test had been accepted for procurement through the WHO Emergency Use Assessment and Listing procedure, which requires extensive validation [33]. This illustrates the importance of reference laboratory capacity. Virus neutralisation is still considered the most specific flavivirus serology test, although cross-reactivity can be observed in patients with other flavivirus infections and research is ongoing to develop more specific assays [10,31,32,34]. Broad implementation of this confirmatory test in EU/EEA national reference laboratories is, however, expected to increase the reliability of serology results in returning travellers as the flavivirus background (in particular dengue) in European travellers is likely to be low.

Molecular testing, having capacity/capability to diagnose ZIKV, relied in 21 of 44 laboratories only on commercial assays, in 14 laboratories only on in-house testing and in nine laboratories on both commercial and in-house testing. Only one (Lanciotti et al. [25]) of the two in-house tests that were most frequently used, was recommended based on in silico analysis [10] and in an independent comparative study of ZIKV molecular tests [35], raising possible concerns about the performance of diagnostics in some laboratories. In November 2016, the ECDC-funded emerging viral disease laboratory expert network (EVD-LabNet [36]) organised an external quality assessment (EQA) for member laboratories which included the majority of the national reference laboratories with ZIKV diagnostics that participated in this questionnaire. The results from such an EQA will provide points of improvement to the individual laboratories [37].

For an adequate choice of which type of test to use and for correct interpretation of test results, a defined set of information on the patient history is essential. As shown for other diagnostics, the lack of information provided by clinicians requesting the diagnostics proved to be a major gap [24]. Although the date of sampling was given most often (40/44 scored ‘often’ or ‘always’), this information is hardly meaningful without an indication of the first day of illness (only scored in the highest categories by 27 laboratories). Reliable interpretation of ZIKV serology is impeded without information on previous flavivirus infections or vaccinations that was often/always available in only 17 of 44 laboratories. Reimbursements rules for the diagnostic tests differ between countries (data not shown). If the state covers the costs for the diagnostic tests, provision of necessary background information can be mandatory.

The overall ZIKV diagnostic capacity in the 30 EU/EEA countries appeared to be sufficient, given the total number of reported requests vs the indicated capacities. At country level, the extent of under/overcapacities varied and were complicated by the fact that laboratories with certain ISO accreditations are bound by strict regulations when sending a surplus of samples to a backup laboratory. The availability of validation materials, positive controls and personnel were indicated as the main challenges for implementation of ZIKV diagnostics in the reference laboratories of the 30 EU/EEA countries. That 15 of 44 laboratories indicated the cost of commercial tests as an obstacle for test implementation and the large dependency of the laboratories on commercial assays, while five of 44 laboratories did not receive funding for development and/or implementation of in-house tests, illustrate an Achilles’ heel in the preparedness for emerging infections and needs careful consideration when defining strategies to strengthen laboratory preparedness and response for future outbreak situations.

## Conclusion

While the coverage and capacity of ZIKV diagnostics in EU/EEA national reference laboratories were observed to suffice in May 2016, the assessment of the quality and needs indicated several crucial points of improvement that will need support at national and EU/EEA level. All reference laboratories should seek to have a relevant ISO accreditation. Awareness and facilitation of (temporary) acceptance of laboratory accreditation for novel diagnostics implemented in emerging situations is required, although it is currently hampered by lack of availability of well-defined validation panels. Improved access is required to controls and validation panels for both molecular and serological tests. Pending the development of more specific serology tests, a broader implementation of the current most specific neutralisation tests (PRNT, VNT) is desirable, ideally in a comparative setting with other relevant flaviviruses. Increased awareness is needed among clinicians to provide all necessary background information, and systems should be implemented that assure provision of necessary interpretation data. National and/or EU contingency funding should be established to ensure adequate and robust laboratory preparedness and response.

## Notes

**Members of the ZIKV reference laboratory group:** Austria: Stephan Aberle, Department of Virology, Medical University of Vienna, Vienna; Belgium: Marjan Van Esbroeck, Institute of Tropical Medicine, Antwerp; Bulgaria: Iva Christova, National Center of Infectious and Parasitic Diseases, Sofia; Croatia: Tatjana Vilibic-Cavlek, Croatian National Institute of Public Health, Zagreb; Cyprus: Jan Richter, The Cyprus Institute of Neurology and Genetics, Nicosia; Czech Republic: Hana Zelena, Institute of Public Health, Ostrava and Helena Jirincova, The National Institute of Public Health, Prague; Denmark: Anders Fomsgaard, Statens Serum Institut, Copenhagen; Estonia: Rita Peetso; Laboratory of Communicable Diseases, Health Board, Tallinn; Finland: Anne J Jääskeläinen, Helsinki University Hospital, HUSLAB, Helsinki; France: Segolene Brichler, Avicenne Hospital, Bobigny and Christine Bigaillon, Begin Military hospital - medical laboratory, Paris and Marina Illiaquer, Centre hospitalier universitaire de Nantes, Nantes and Salim Gaba, entre hospitalier de Perpignan, Perpignan and Geraldine Gonfrier, Centre hospitalier universitaire de Nice, Nice and Christophe Ramière, Institut des Agents infectieux, Hôpital de la Croix-Rousse, Lyon and Isabelle Leparc-Goffart, Institut de Recherche Biomédicale des Armées, Centre National de Référence des arbovirus, Marseille and Jean-Dominique Poveda, Laboratoire Cerba, Saint-Ouen-L'Aumone and D. Hober, Laboratoire de virologie, Centre Hospitalier Regional Universitaire de Lille, Lille and H. Fleury, Laboratoire de virologie, Centre Hospitalier Universitaire de Bordeaux, Bordeaux and Samira Fafi-Kremer, Laboratoire de Virologie, Hopitaux Universitaires de Strasbourg, Strasbourg and V. Foulongne, Centre Hospitalier Universitaire de Montpellier, Montpellier and Cécile Henquell, Centre hospitalier universitaire de Clermont-Ferrand, Clermont-Ferrand and Nicolas Leveque, Virology and Mycobacteriology Department, Centre hospitalier universitaire de Poitiers, Poitiers and Marie Josee Carles, Virology departement, Centre hospitalier universitaire de Nimes, Nimes and JM. Mansuy, Virology department, Centre hospitalier universitaire de Toulouse, Toulouse; Germany: Jonas Schmidt-Chanasit, Bernhard Nocht Institute for Tropical Medicine, Hamburg and Andreas Nitsche, Robert Koch Institute, Berlin; Greece: Anna Papa,National Reference Centre for Arboviruses, Aristotle University of Thessaloniki, Thessaloniki and Andreas Mentis, Hellenic Pasteur Institute, Athens; Hungary: Mária Takács, National Center for Epidemiology, Budapest; Iceland: Arthur Löve, Department of Virology, Landspitali- National University Hospital, Reykjavik; Ireland: Jeff Connell, National Virus Reference Laboratory, University College Dublin, Dublin; Italy: Giulietta Venturi, Istituto Superiore di Sanità, Rome; Latvia: Jelena Storoženko, Latvian Centre of Infectious Diseases, Riga East University Hospital, Riga; Lithuania: Algirdas Griskevicius, National Public Health Surveillance Laboratory, Vilnius; Luxembourg: Matthias Opp, Laboratoire National de Santé, Dudelange; Malta: Christopher Barbara, Mater Dei Hospital, Msida; Netherlands: Chantal Reusken, Erasmus University Medical Center, Rotterdam and Johan Reimerink, National Institute for Public Health and the Environment, Bilthoven; Norway: Susanne Gjeruldsen Dudman, Norwegian Institute of Public Health, Oslo; Poland: Katarzyna Pancer, National Institute of Public Health-National Institute of Hygiene, Warsaw; Portugal: Maria João Alves, National Institute of Health Dr. Ricardo Jorge, Lisbon; Romania: Cornelia S. Ceianu, Cantacuzino National Institute of Research & Development, Bucharest; Slovak Republic: Elena Tichá, Public Health Authority of the Slovak Republic, Bratislava; Slovenia: Tatjana Avšič Županc, Institute of Microbiology and Immunology, Faculty of Medicine, University of Ljubljana, Ljubljana; Spain: Maria Paz Sanchez-Seco, Instituto de Salud Carlos III, Madrid; Sweden: Thomas Tolfvenstam, Public Health Agency of Sweden, Solna; United Kingdom: Emma Aarons, Rare and imported pathogens laboratory, Public Health England, Porton Down.
